# *Homo Developmentalis*: An evolutionary proposal relevant for child and adolescent mental health

**DOI:** 10.3389/frcha.2022.940827

**Published:** 2022-11-02

**Authors:** David Cohen, Axel Baptista

**Affiliations:** ^1^Service de Psychiatrie de l'Enfant et de l'Adolescent, GH Pitié-Salpêtrière Charles Foix, APHP.SU, Paris, France; ^2^Institut des Systèmes Intelligents et de Robotique, Sorbonne Université, ISIR CNRS UMR 7222, Paris, France; ^3^Institut Jean-Nicod, Département d'Études Cognitives, INSERM U8129, École Normale Supérieure, PSL Research University, Paris, France

**Keywords:** development, child and adolescent, evolution, mental health, externalizing disorder

## Introduction

*Homo Developmentalis* proposes an evolutionary perspective on child development and psychopathology. It reflects the dynamics of recent changes in *Homo Sapiens*: from hunter-gatherer to megalopolis city dweller; from attachment to the tribe to equality among all; from the discovery of writing to that of artificial intelligence. All of these changes show the importance of early environmental interactions and contexts for the baby's development. They have favored during evolution bidirectional interactions between the brain and environment creating a complex interplay. By coupling this evolutionary perspective to a categorization of the environmental factors into micro (e.g., alcohol during pregnancy, child abuse) and macro (e.g., cultural background, group affiliation), we discuss in this essay its relevance for child and adolescent mental health. To do so, we focus on two psychopathologies – externalizing disorders and borderline personality disorders – exploring causality from an evolutionary developmental perspective and suggesting that life history strategies may correspond to intermediate normal phenotypes.

## Characteristics of *Homo Sapiens* in the mammal order

*Homo Developmentalis proposed as a* term and concept that reflects the dynamics of recent changes that *Homo Sapiens* has encountered [1]. These recent changes cannot be explained by evolutionary changes relying on classic natural selection theory (e.g., pressure on our genes). We have ~22,000 genes, which is an insufficient number to organize a social thought as accomplished as that of humans [2]. Genetics is not the only mode of transmission in humans since many non-genetic factors also associated with issues of inheritance or heredity have been described [3]. Novel perspectives regarding evolution consider that interactions with the environment are also crucial [4].

Let us recall some of the characteristics of the human species whose emergence has been favored by evolution. Physically, human morphology when compared to that of the non-human primates (monkey) presents many distinctions: standing position, hand and foot morphology, and disappearance of facial and body hair, which probably favors taking in information in terms of facial expression, and noticeably increased brain size [5]. The counterpart of this standing position and the growth of the brain forced the delivery of babies to advance because of the physical limits of the pelvic bone constitution (estimations suggests that deliveries should take place at approximately 21 months and not at 9 months). The brain is particularly immature at birth, continues to have fetal brain characteristics and develop after birth. The human baby is therefore in a position of great dependence; it develops slowly, cannot move on its own, nor can it cling to its mother like its primate cousins. For some authors, this necessary motor separation between the baby and its mother in the first humans may have favored the appearance of an audio channel to communicate the baby's distress (e.g., crying), parental comfort (e.g., motherese), and therefore favor the appearance of language [6].

At the same time, communities authorized a sharing of food and techniques to obtain nutrients that probably promoted both learning and mobility. Moreover, unlike non-human primates, gestational gaps in humans shorten as the lifespan increases. Thus, despite the increase of infant's dependence (compared to non-human primates), this change favors the presence of fathers, large families, and mutual aid among family members; ultimately, this gestational gap may promote learning by proximity and imitation, transferring behaviors and cultural heritage. Indeed, inheritance and transmission cannot be reduced to genes. There are at least three other modes of transmission: epigenetic transmission, behavioral transmission, and symbolic/cultural transmission [3]. The latter has extremely powerful effects, such as cultural biases and stereotypes associated with social affiliation groups [7]. The affiliation group defines a territory as a security zone. During evolutionary time, the affiliation group was the small group in which each individual evolved. Despite very early abilities showing infant's altruism, rejection of the other (or racism) has long been an evolutionary advantage because it has fostered cohesion within an affiliated group such as the tribe for *early Homo*. During our cultural evolution and in parallel with changes in our lifestyles (overcrowding in megacities), we have developed values of humanism and tolerance that have enabled changes. However, the stereotypes remain powerful. Thus, amorous behaviors are mainly experienced in affiliation groups. In other words, humans are naturally racist. Adopting opposing cultural values during development runs counter to the natural order [8]. These changes during evolution have also profoundly modified development (schooling and the acquisition of reading) and social interactions (learning civic principles and human rights). Thus, based on all aforementioned changes, we have proposed to call contemporary man *Homo Developmentalis*. This evolution also continues in our contemporary world with the observation of a humanistic metamorphosis of European civilization.

## The child's environment: An ecosystem in interaction

From the above, we understand that the child develops in an environment of extreme variety. From a systemic perspective, environmental factors are considered in a gradient of distance from the child [9]. At the most proximal, we isolate the contribution of toxic environmental factors that will have a direct effect on brain development during pregnancy or the first years of life (e.g., alcohol, air pollution). The second type of environmental factor is the microenvironment, which includes individual and family factors (e.g., abuse). The third type of environmental factor is the macroenvironment. In the latter, a further distinction is made between the exosystem or extended environment (e.g., school, peers, extended family) and the macrosystem or social frame of reference (e.g., culture, law, religion) [10]. At the very beginning of life, a baby is immediately part of this ecosystem with few emerging functions with which to interact (e.g., imitation, gazing, “speech turn”). The quality of early interactions is decisive [11–13]. The baby's brain is a sponge toward environmental stimulation and cues. Indeed, the environment can act on the brain and influence its future development through many mechanisms: statistical learning [14], stress regulation [15], epigenetic mechanisms and transgenerational transmission of behavioral patterns [16], gene/environment interactions [17], synchronous interactive signals taking on the valence of a social signal amplified by certain hormones (e.g., oxytocin) [18], and cultural recycling of brain areas to promote learning [19].

Early interactions are a cornerstone of the modern evolution of *Homo sapiens* [20]. Compared to non-human primates, modern humans have late development and an intense need for social contact. In addition to meeting the basic needs of infants (e.g., food) or being a condition for early learning (e.g., mother tongue), the quality of the parent–child relationship plays a role in the child's social, emotional and cognitive development. Interactions between infants and their partners occur on four different levels: behavioral, embodied, affective, and imaginary. They contribute to the emergence of intersubjectivity and the construction of the self [21]. In the majority of situations, the result is a child with a harmonious development who, upon entering school, presents a certain number of qualities that are protective *vis-à-vis* the later vicissitudes of life: feelings of inner security, positive self-esteem, and a capacity for cognitive inhibition and affective resilience.

## Dialogue between learning and environment: The effect of culture

The learning of language explains a great deal about how the environment contributes to the intimate development of the brain. It is spontaneous for oral language and cultural for written language. Language is one of the most fabulous and complex human cognitive abilities. It provides us completely unique possibilities of reflection and communication simultaneously at the cognitive and interactive levels. However, it is wrong to imagine that animal species are not capable of communication since we find capacities for perceptual categorization in monkeys and birds, capacities for learning species-specific vocalizations in monkeys and birds, and a form of motor babbling has even been described in monkeys [22].

Oral language is a function that arises in humans from early exposure to a language. A human cannot develop this function if, as a baby, he or she is not spoken to. At the same time, this development occurs over time and does not require formal learning. In this regard, it differs from learning written language or reading, which requires being taught at home or at school. Babies deprived of language interactions during infancy cannot develop oral language. Thus, we can see that this function in humans arises in the dynamics of early language interactions and does not occur on a tabula rasa or an exclusive genetic determinism [14]. One of the elements that can be identified early is the specialization for the phonemes of the mother tongue at the age of 6 months. At this age, we see that babies are more attentive to the phonemes of their language and that, moreover, in their vocalizations, they will begin to use the specific vocalizations of their mother tongue much more frequently. This specialization in the phonemes of a language responds to statistical learning rules. The child explores the frequency properties of language to which he is exposed. This early experience will constrain the perceptual system at the neural level. Mere exposure to language is insufficient, and alongside the aspects of language perception and production, there is also a dimension of social and emotional interaction that strongly influences this learning [14].

Learning to read is also an extraordinary example of cerebral plasticity and an illustration of the way in which education can “recycle” cerebral structures and modify them for the appearance of a new function. From an evolutionary point of view, it is a recent cultural learning (~6,000 years ago). Without specific teaching whether at school or at home, the child does not learn to read. Learning to read involves visual, auditory and motor functions. With most adult readers, these tasks are fully automated. Many factors can influence learning to read, such as oral language skills, context and psychosocial support, educational strategies, and certain cognitive or developmental aspects (attention, phonological skills, visual-spatial discrimination, cognitive processing speed), which in some cases will lead to dyslexia [23]. Even in recent years when exclusively biological and genetic views have been dominant, studies have shown the decisiveness of cultural and social factors [24]. School education modifies the visual cortical areas and those of language according to a gradient proportional to the intensity of this learning and its practice [25].

**Figure 1:**
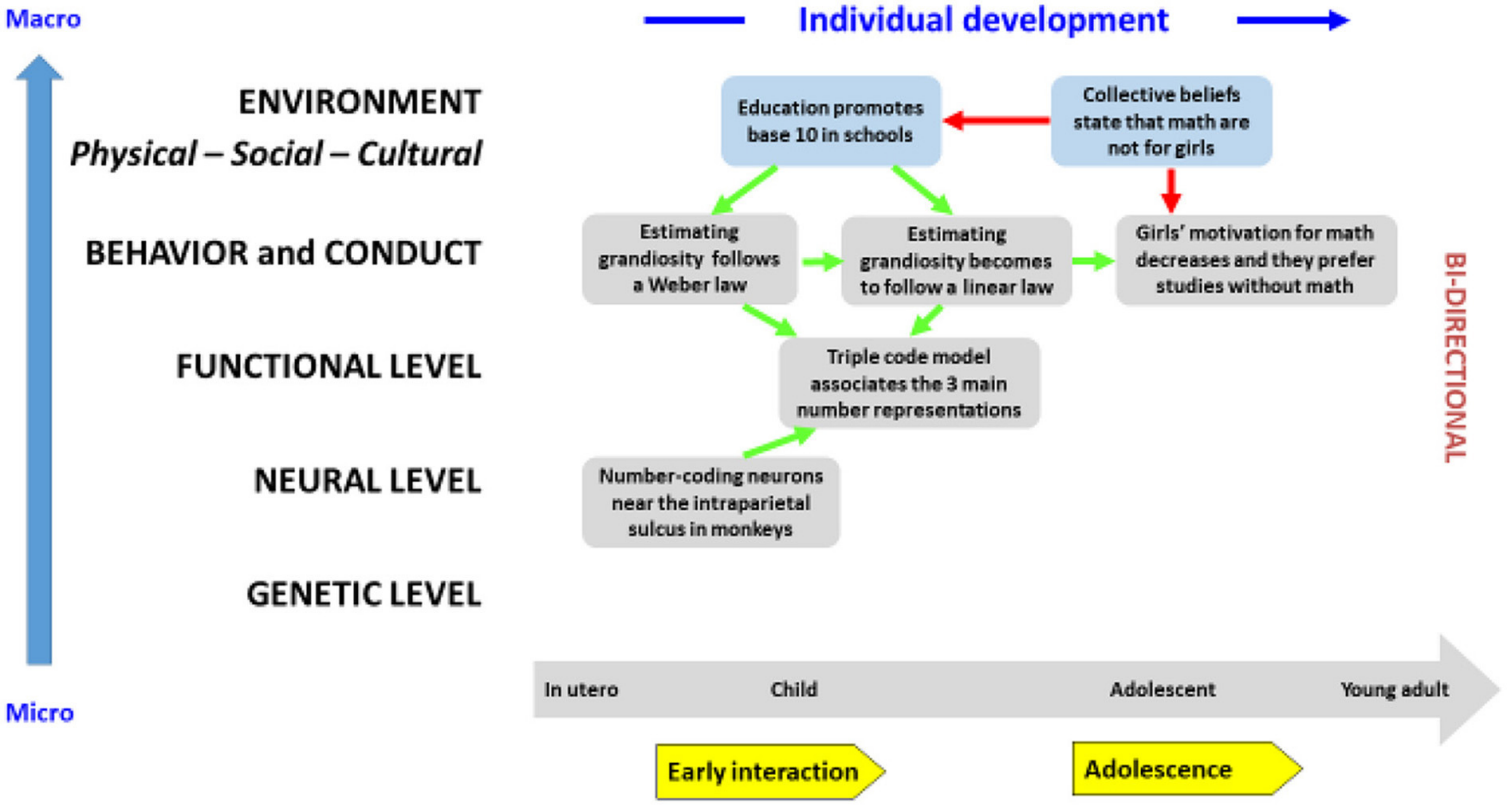
An Evo-Devo model for mathematics.

The development of mathematics (Figure 1) gives us an example of cultural constraints on innate aptitudes involving all levels of the ecosystem. Concerning the acquisition of numeration, the adult is able to manipulate different representations that can be symbolic (Arabic numeral and written word) or non-symbolic (estimate of size or subitizing). From an evolutionary point of view, humans are not the only animal to be able to compare sizes, since we can find this ability in monkeys. It responds to a logarithmic law, i.e., it is particularly efficient for the most distant quantities. The baby also has this estimation of quantity. This very early knowledge in the baby is also present in adults who have not received any education or training in mathematics. In educated adults, the representation in Arabic numerals becomes very strongly associated with the non-symbolic processing of quantity, which ends up creating a completely automated second-order intuition [26]. In Western societies, this second order of intuition follows a linear representation in base 10 favored in schools. Intensive mathematics education therefore transforms the initial logarithmic representation into a linear representation. It is a fine example of cultural influence (here, the exosystem represented by the school) on a faculty, which is not only increased during development but also transformed in its very nature [26]. Learning math skills also gives us an example of the importance of the macrosystem because of very strong gender stereotypes toward mathematics (“girls are less good at math than boys”). In all Western societies, the vast majority of engineers and mathematicians are men (e.g., only one woman has received the Fields Medal since its creation), with the exception of Korea and Singapore that do not value these stereotypes. In other Western societies, these stereotypes are transmitted by families and teachers. Since the launch of programs to combat these stereotypes, we have observed that the performance of girls in math tends to approach that of boys in societies where gender equality is promoted [27].

## The development of executive functions and secure attachment

Executive functions include working memory, attention and inhibition skills, and cognitive flexibility. These are determining cognitive functions for learning but also for adapting to the world and for most complex cognitive functions such as reasoning or problem solving. However, these do not have a continuous progression until adulthood, and they depend on the maturation of the prefrontal cortex. The prefrontal cortex is the last region of the brain to evolve and takes the longest to reach full maturity. Thus, in children, working memory is quickly of good quality, but inhibitory control is much less so [28]. This inhibitory control involves being able to control one's attention, behavior, thoughts and/or emotions to overcome strong internal stimulation or external lures and achieve what is expected of oneself. In other words, it is a question of inhibiting the first heuristics to enter into new learning [29]. It is only around the age of six that the child has enough capacity for inhibition. It is for this reason that in most Western countries, schooling begins at this age. For some authors, the epidemic of children with attention deficit hyperactivity disorder (ADHD) is partly linked to changes in schooling methods and current societal constraints *vis-à-vis* young children [2]. However, it is now accepted that education influences the development of inhibition capacities as do many other environmental factors, which not only affect the development of executive functions but also are risk factors for externalized disorders (see below).

If the development of executive functions is a pivot of early development on a cognitive level, its counterpoint on the psycho-affective level is the development of attachment. If we take an evolutionary perspective on how affiliation relationships and the construction of attachment behaviors are organized, we can better understand the following significant changes [30]. (1) During evolution, we move from conditions of attachment based on promiscuity, olfaction and nesting in rodents to conditions of attachment based on exclusivity, multisensoriality, partnership and culture in humans. Indeed, in humans, an evolution of parental behavior has allowed the phenomena of synchrony to overcome the olfactory and touch senses, thus increasing the distance while keeping the link possible. (2) Over the course of evolution, positive emotional investment in affiliate bonds and in building a secure attachment have become more important. In rodents, the negative emotional valence is prevalent and constitutes a determining element of alarm for the sharing of danger and the response to stress. In humans, the repertoire of so-called negative emotions is much more subtle, but these remain associated with fear and danger or cultural avatars (taboos, prohibitions). However, we see in monkeys that facial expressions exist and can be invested in a mode of non-verbal communication between conspecifics and even with different meanings depending on the culture of the group within the same species [31]. In humans, the fact of being able to increase the distance of early bonds is contemporaneous with the appearance of a verbal/audio channel that from the start of early mother-baby relationships is an intentional mode of communication. The distress signal then becomes the crying of the baby [6]. At the same time, the mother will develop a way of talking to the baby called motherese, which is very rich in emotional valence [32]. Early exchanges are therefore not only multisensory but also emotionally embodied positively and/or negatively.

At a less intimate level of interaction between individuals, we can also look at how interest in others (or empathy in its broadest sense) may have become more complex during evolution. We readily distinguish between emotional empathy, which corresponds to concern for others and is found in mammals and perhaps birds, and cognitive empathy, which concerns certain large mammals and primates. There is also a tendency to sometimes oppose competitive behaviors and support cooperative behaviors at the level of 2-on-2 interactions and within or between social groups. However, it is clear that this behavioral opposition is also an emotional valence opposition that is positive for cooperation and negative for competition. Likewise, the way in which group ties are formed through identity, political, religious, and artistic representations can also be understood through the emotional valence associated with them. In an emotionally negative way, the binding and the cohesion of the group arise from fear of the other in the political (nationalism and populism), religious (fundamentalism) or artistic (art qualified as primitive or degenerate) spheres, or positively when binding and cohesion relate to openness to others and fulfillment through sharing and respect for differences. To put it another way, emotional investment promotes all levels of ties (therefore of interaction) that humans and human societies can develop: affiliation, attachment, love, transgenerational family, and social and cultural identity.

## Relevance for child and adolescent mental health

### The “life history style” theory

*Homo sapiens* is part of the long evolutionary history mentioned above. The Life History Theory is part of this perspective, and makes it possible to account for the inter-individual differences in the developmental timing and expression of evolutionarily influenced traits. It addresses why and how individuals distribute their limited metabolic resources between growth, survival and reproduction, which are three major challenges of evolution. The longer developmental period of *homo developmentalis* allows him to more flexibly adapt to environmental conditions. Life History Theory is thus a particularly relevant framework to explain inter-individual differences in contemporary humans' traits that are highly influenced by the environment, as mentioned above.

Depending on the level of adversity experienced during infancy and childhood (harsh and unpredictable environment *vs*. soft and predictable environment), the theory distinguishes two adaptive styles called “fast” and “slow” characterized by a series of oppositions both at the level of personality traits as well as mental and physical health risks. The fast style is characterized by greater motivation for short-term benefits, frequent insecure attachments, earlier puberty and sexuality, an unstable emotional life, more anxiety and depression pathologies, and more cardiovascular issues. In contrast, the slow style is characterized by opposite cues. These styles, which are not pathological themselves, illustrate how, from an evolutionary point of view, individuals subjected to more adversity will favor adaptive methods that make it possible to respond to the immediate dangers perceived as more numerous and to expose them to greater risk of learning aggressive behaviors [33].

### Contribution for conditions with aggressiveness

From birth, a child knows how to show his displeasure even before he becomes angry. There is a continuum between his or her reactions to lack, frustration and the manifestation of more or less aggressive demands regarding asking, claiming, and demanding. The first directly aggressive behaviors occur at the end of the second year and during the third year. Previously, toddlers may present rage reactions. At ~2–3 years, the child frequently adopts opposing or angry behaviors. At this time, he or she attacks, claws, pulls hair, and bites children of his own age during play. These reactions fade at approximately 4 years old: the child expresses his aggressiveness verbally but no longer in behaviors. At this stage, the child's aggressive fantasies are often rich and numerous, as evidenced by his or her games, and at the same time, dreams of anxiety and aggression make their appearance. However, violence is minimal as inhibitory processes help the child to socialize [23].

**Figure 2:**
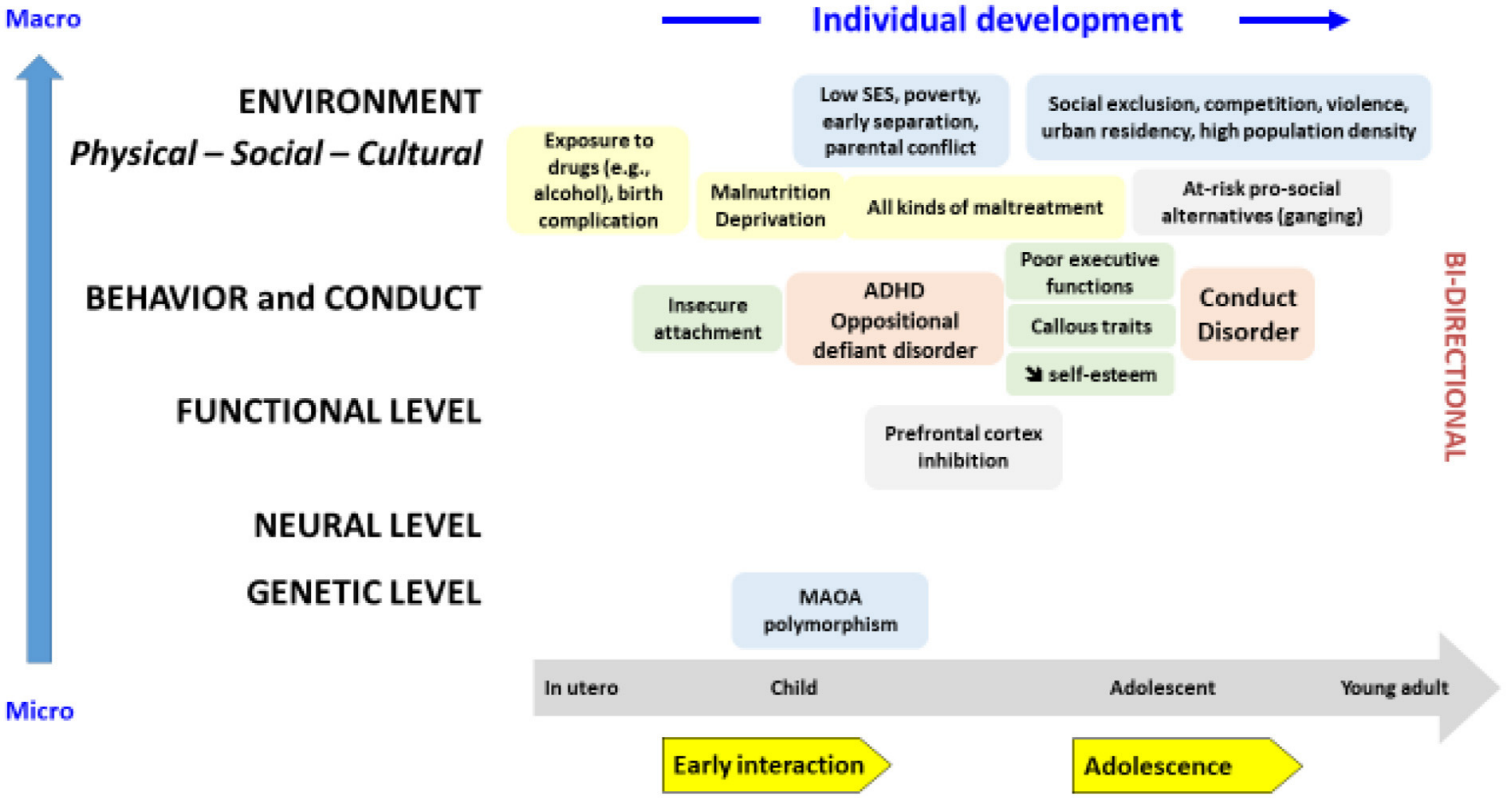
An Evo-Devo model for externalizing disorders.

Some children continue to be violent, hitting their classmates, even adults or their parents, breaking other people's things or their own. These children often belong to the fast style of the “life history style” theory and may enter into violent anger or even real reactions of rage [34]. They tend to use others and especially their parents as mere instruments placed at their disposal: they do not tolerate any delay in the satisfaction of their requests. Sometimes this attitude is selective, occurring only in the presence of certain people: one or the other parent or teachers. To a greater degree, the reaction of intolerance to frustration can appear for the most minimal reasons even outside of any relationship with a person. The sequence of anger/agitation/kicking or punching in all directions/final self-aggression shows the importance of aggressive impulses, the poor distinction between the self and the external world, and the difficulties of emotional regulation and cognitive inhibition. In mental health nosography, pathological aggressiveness as a symptom appears in several clinical pictures. For some children, aggression/irritability can be considered a manifestation of depression; as a cardinal symptom, aggression appears mainly in childhood externalizing disorders: ADHD, oppositional disorder with defiant behavior (ODD), conduct disorder (CD) and disruptive mood dysregulation disorder (DMDD). Figure 2 summarizes how to determine these main pathologies during development in the context of the developmental and micro/macro perspectives mentioned above. In terms of age of onset, pathologies are part of a developmental agenda, with binomial ADHD/ODD appearing in childhood, then as CD in preadolescence, and finally as antisocial personality in adults. Transitions are not systematic during development and depend on modulating factors such as level of impulsivity, aggression and hyperactivity; lack of empathy or prosocial emotion; capacity for inhibition or guilt; low self-esteem, insecure attachment, or difficulty with emotional regulation; and very importantly environmental factors such as hostile critical parenting. Environmental factors are numerous, diverse and affect all micro/macro levels [35]. It should be noted that the causal valence of most of them is non-specific since several of them are associated with an increased risk of learning aggression pathologies but also of anxiodepressive, borderline or neurodevelopment pathologies [36].

### Contribution to borderline psychopathology

Borderline personality disorder (BPD) is a severe and frequent disorder in adolescents characterized by a pervasive pattern of instability affecting impulse control, emotional regulation, cognitive processing, sense of self and agency and interpersonal relationships. Patients' personal histories are often marked by stressful or traumatic experiences, often repeated. Clinical signs of the disorder include both chronic and acute features, with acute features being mostly triggered by acute stressful situations. Such features include transient cognitive distortion, intense anger, uncontrollable impulsivity, and self-harm behaviors (including suicide) and contribute to the burden of the disease [37]. Many aspects, including epidemiological, clinical, and physiological aspects, contribute to the relationship between BDP and stress as an adaptation to adversity. Although the pathogenesis of BDP remains uncertain, the literature reflects a specific biological and neural pattern of altered stress perception and regulation in BPD.

**Figure 3:**
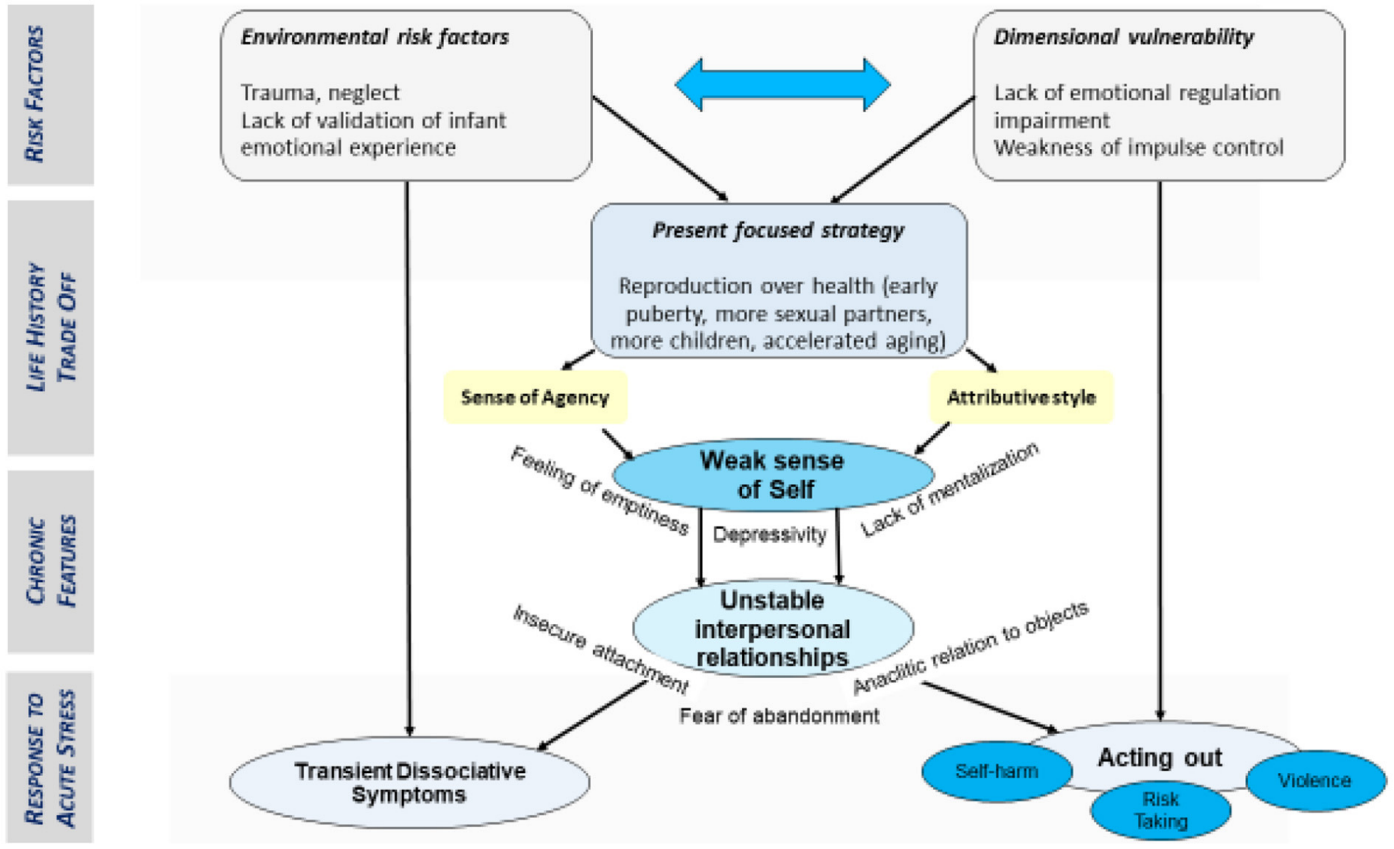
Self-disturbances, adverse life conditions and borderline psychopathology.

Figure 3 addresses the poorly characterized relation between self-disturbances and adverse life conditions encountered early in life in BDP. We highlight the potential relevance of “life history style” theory (the aforementioned major framework in evolutionary developmental biology) to make sense of this association. We put forward the idea that the effect of early life adversity on BPD symptomatology depends on the way individuals trade their limited resources between competing biological functions during development. The available literature supports this hypothesis, as BDP seems to share the main characteristics found in the fast style of the “life history style” theory [38]. However, in contrast to externalizing disorders such as conduct disorder, in which impulse control and executive functions may be altered, it appears in BDP that interpersonal hypersensitivity, insecure attachment, identity disturbance, emotion dysregulation are also significant psychological dimensions. To add another layer of complexity, some authors argue that emotional dysregulation is in fact an affective instability or an issue related to inhibitory control of affective impulse.

## Conclusion

*Homo Developmentalis* proposes an evolutionary perspective on child development and psychopathology. The central argument and perhaps the originality is to debunk a deterministic and neuroconservative vision of development by showing how bidirectional interactions between the brain and environment create a complex interplay, by coupling this evolutionary perspective with a categorization of the environmental factors into micro (e.g., alcohol during pregnancy, child abuse) and macro (e.g., cultural background, group affiliation). In this essay, I focused on two psychopathologies—externalizing disorders and borderline personality disorders—exploring causality from an evolutionary perspective and suggesting that the “life history style” theory may be an intermediate normal phenotype. These examples respect a micro-macro gradient of environment that I illustrated on a case-by-case basis.

## Author contributions

DC designed the challenge, formulated the hypothesis, and wrote the first draft and figure. DC and AB reviewed the literature. AB revised the final manuscript. Both authors contributed to the article and approved the submitted version.

## Conflict of interest

The authors declare that the research was conducted in the absence of any commercial or financial relationships that could be construed as a potential conflict of interest.

## Publisher's note

All claims expressed in this article are solely those of the authors and do not necessarily represent those of their affiliated organizations, or those of the publisher, the editors and the reviewers. Any product that may be evaluated in this article, or claim that may be made by its manufacturer, is not guaranteed or endorsed by the publisher.
